# Effects of an Immersive Virtual Reality Intervention on Pain and Anxiety Among Pediatric Patients Undergoing Venipuncture: A Randomized Clinical Trial

**DOI:** 10.1001/jamanetworkopen.2023.0001

**Published:** 2023-02-16

**Authors:** Cho Lee Wong, Kai Chow Choi

**Affiliations:** 1 The Nethersole School of Nursing, Faculty of Medicine, The Chinese University of Hong Kong, Hong Kong

## Abstract

**Importance:**

Venipuncture is one of the most painful and distressing procedures experienced by pediatric patients. Emerging evidence suggests that providing procedural information and distraction using immersive virtual reality (IVR) may reduce pain and anxiety among children undergoing needle-related procedures.

**Objectives:**

To examine the effects of IVR on reducing the pain, anxiety, and stress experienced by pediatric patients undergoing venipuncture.

**Design, Setting, and Participants:**

This 2-group randomized clinical trial recruited pediatric patients aged 4 to 12 years undergoing venipuncture from a public hospital in Hong Kong between January 2019 and January 2020. Data were analyzed from March to May 2022.

**Interventions:**

Participants were randomly allocated to an intervention (an age-appropriate IVR intervention offering distraction and procedural information) or a control (standard care only) group.

**Main Outcomes and Measures:**

The primary outcome was child-reported pain. Secondary outcomes included child-reported anxiety, heart rate, salivary cortisol, length of procedure, and satisfaction of health care professionals with the procedure (rated on a 40 point scale, with higher scores indicating greater satisfaction). Outcomes were assessed 10 minutes before, during, immediately after, and 30 minutes after the procedure.

**Results:**

A total of 149 pediatric patients were recruited, with 86 female patients (57.7%) and 66 patients (44.3%) diagnosed with fever. Compared with the 74 participants in the control group (mean [SD] age, 7.21 [2.49] years), the 75 participants in the IVR group (mean [SD] age, 7.21 [2.43] years) reported significantly less pain (β = −0.78; 95% CI, −1.21 to −0.35; *P* < .001) and anxiety (β = −0.41; 95% CI, −0.76 to −0.05; *P* = .03) immediately after the intervention. Health care professional satisfaction in the IVR group (mean [SD] score, 34.5 [4.5]) was significantly higher than that in the control group (mean [SD] score, 32.9 [4.0]; *P* = .03). Moreover, the length of venipuncture procedure in the IVR group (mean [SD] duration, 4.43 [3.47] minutes) was significantly shorter than that in the control group (mean [SD] duration, 6.56 [7.39] minutes; *P* = .03).

**Conclusions and Relevance:**

In this randomized clinical trial, integrating procedural information and distraction in an IVR intervention for pediatric patients undergoing venipuncture significantly improved pain and anxiety in the IVR group compared with the control group. The results shed light on the global trends of research on IVR and its clinical development as an intervention for other painful and stressful medical procedures.

**Trial Registration:**

Chinese Clinical Trial Registry identifier: ChiCTR1800018817

## Key Points

**Question:**

Compared with standard care, do distraction and procedural information provided through immersive virtual reality (IVR) improve pain among pediatric patients undergoing venipuncture?

**Findings:**

In this randomized clinical trial of 149 pediatric patients undergoing venipuncture, an IVR intervention significantly improved patient-reported pain.

**Meaning:**

These findings suggest that IVR was more effective than standard care in improving pain among pediatric patients undergoing venipuncture.

## Teaser

This randomized clinical trial examines the effects of immersive virtual reality on reducing the pain, anxiety, and stress experienced by pediatric patients undergoing venipuncture.

## Introduction

Venipuncture is one of the most commonly performed needle-related procedures in hospitalized pediatric patients.^1,2,3^ Pediatric patients undergoing this procedure, particularly those aged 4 to 12 years, often display high levels of pain and anxiety.^4^ Evidence has also shown that procedural anxiety is positively associated with increased self-reported pain.^5^ However, the effect of pharmacological and nonpharmacological management of procedural pain and anxiety remains understudied.^6,7^ Poorly managed procedural pain and anxiety can have short- and long-term consequences to patients, increase the time and resources to perform the procedure, and decrease the health care professionals’ satisfaction with the procedures.^6,7,8,9,10,11^ Therefore, clinical guidelines emphasize the importance of managing procedural pain and anxiety in pediatric patients.^12^

A distraction intervention is the most frequently used nonpharmacological intervention in clinical settings to manage procedural pain and anxiety in pediatric patients undergoing needle-related procedures.^13,14,15^ Compared with other nonpharmacological interventions, such as hypnosis and cognitive behavioral therapy, distraction is easier to implement, and it does not require specialized training.^13,14,15^ Previous studies have used various types of distractors, such as watching cartoons, playing with toys, or soothing by adults, to distract pediatric patients during procedures.^13^ In particular, age-appropriate distractors appear to enhance the effectiveness of pain management.^15,16^ Two studies^17,18^ found that distraction combined with procedural information yields better results. Nevertheless, offering age-appropriate distraction and procedural information at the same time is difficult. Furthermore, previous distractors have failed to completely pull a patient’s attention away from the needle, which is the most anxiety-provoking scene during such procedures.^6,7^ Therefore, identifying a distractor that can totally block children’s attention to pain and anxiety stimuli is of utmost importance.

Emerging evidence suggests that immersive virtual reality (IVR) interventions can help completely distract patients during medical procedures.^19,20^ IVR provides a form of human-computer interaction in which users engage in a computer-generated 3-dimensional virtual world through a head-mounted display.^19^ IVR interventions can be used anytime and anywhere without additional manpower. VR scenarios can also be customized to provide users with health information. Several studies have demonstrated that IVR intervention significantly improved pain and anxiety among pediatric patients undergoing needle-related procedures.^21,22,23,24^ However, they are limited by small sample sizes, patients with a wide age range (5 to 19 years), a lack of evaluation on health care professional satisfaction, and nonuse of age-appropriate VR scenarios.^21,22,23,24^ Most importantly, no studies have simultaneously adopted IVR to provide procedural information and complete distraction for pediatric patients undergoing venipuncture. Guided by Melzack and Wall’s gate-control theory^25^ and Lazarus and Folkman’s stress and coping theory,^26^ we have developed an IVR intervention to address these literature gaps. The theoretical framework has been described previously.^27^ In brief, we posit that IVR offers visual and auditory stimuli that can effectively close the patients’ pain gate^25,28^ while the procedural information can enhance patients’ sense of control, thereby reducing their anxiety.^29^

The present study aimed to examine the effects of the IVR intervention on pain, anxiety, stress, and length of procedure among pediatric patients undergoing venipuncture. Health care professional satisfaction with the procedures was also examined.

## Methods

### Study Design

This study was a 2-group randomized clinical trial. The detailed protocol is reported elsewhere.^27^ Ethical approval was obtained from the Joint Chinese University of Hong Kong–New Territories East Cluster Clinical Research Ethics Committee. The protocol is provided in Supplement 1. This report follows the Consolidated Standards of Reporting Trials (CONSORT) reporting guideline. Written informed consent was obtained from parents of participating children.

### Setting and Participants

This study was conducted in the pediatric unit of a regional public hospital in Hong Kong. For patients younger than 12 years, venipuncture was performed by a medical intern or medical officer with the assistance of a nurse or patient-care assistant in a treatment room. We included pediatric patients who (1) were aged between 4 and 12 years, (2) planned to undergo venipuncture, and (3) were able to communicate in Chinese and follow instructions. Exclusion criteria were as follows: (1) identified cognitive or learning problems or sensory impairments to pain, (2) identified contact precautions, and (3) history of seizures or motion sickness. Health care professionals participating in the procedure (eg, medical interns and officers, nurses, and patient-care assistants) were also invited to rate their satisfaction with the venipuncture procedure.

The sample size calculation was based on the effect estimated from a previous VR study using the Faces Pain Scale–Revised (FPR-S) as a primary outcome measure.^21^ By using the power analysis software GPower version 3.4, it was estimated that a sample size of 85 participants per group would enable a 2-group randomized clinical trial to detect a between-group difference of 0.8 points on the FPS-R scale, with a pooled standard deviation of 1.84 and 80% power at 5% level of significance. Accounting for an attrition rate of up to 15%, we aimed to recruit 200 pediatric patients, with 100 patients in each group.

### Randomization and Masking

Eligible participants were randomly assigned to the intervention or control groups by using stratified permuted block randomization (block size = 10) in a 1:1 ratio. Randomization was stratified by age group (4-7 and 8-12 years) considering the potential difference in pain-stimulus sensitivity in these 2 developmental stages.^30,31^ A random sequence list of group identifiers was generated by an independent statistician using computer-generated random codes for each age group. These identifiers were placed in a sealed opaque envelope with a serial number. Pediatric patients were allocated based on their age, order of enrollment in the trial, and group identifier contained in the corresponding numbered envelope. Research assistants (RAs), ward staff, patients, and parents were blinded to the group assignment until consent and baseline assessment data were obtained. However, owing to the nature of the intervention, blinding the participants, RAs, and health care professionals was difficult.

### Standard Care and IVR

Patients in the control group received standard care, including explanation of the venipuncture procedure and comforting wordings. Patients in the intervention group received the IVR intervention in addition to standard care. The IVR intervention aimed to instill a sense of control by offering patients procedural information and distracting them from an anxiety-provoking venipuncture scene.^8,29,32^ The details of the intervention have been outlined in the published protocol.^27^ Briefly, the IVR intervention offered 2 age-appropriate VR scenarios through a disposable headset that can be fitted into smartphones. Both scenarios involved a self-designed cartoon character DD who was going to undergo venipuncture. Given that visual stimuli can effectively distract children,^29,33^ the scenario presented screen zooming at various paces, with the cartoon character DD enacting various body movements.^33^ Pastel-tone colors were used to reduce eye strain while providing visual stimulation to the patient. For the scenario presented to patients aged 4 to 7 years, procedural information on why DD required venipuncture was presented using simple words and sentences in a child-centered manner. For patients aged 8 to 12 years, more detailed information—such as why the procedure was performed, what to expect, and how the procedure would feel—was explained, followed by an interactive game in which the user played the role of the doctor and prepared the equipment required for the venipuncture procedure. The eFigure in Supplement 2 includes screenshots of the scenarios.

The RA first provided instruction to patients about how to use the VR equipment and then placed the head-mounted display on the patient’s head, making adjustments to ensure a comfortable and secure fit. Patients were then allowed to watch the age-appropriate scenarios starting 5 minutes before and until the completion of the venipuncture procedure.^21,22^ They were also told that if they experienced any discomfort, such as motion sickness and eye discomfort, the intervention would be discontinued. Meanwhile, the medical officers or interns were invited to synchronize the steps of the venipuncture procedure with the script of VR scenario, so that what was going on in the VR scenario actually happened in reality.

### Measurements

#### Pain Level

The FPS-R was used to assess the pain level of the participants. It is a 0-to-10 scale comprising 6 horizontally arranged cartoon faces with expressions of 0, indicating no pain, to 10, indicating very painful.^34^ This scale has been shown to be reliable and valid for evaluating children’s self-reported pain.^10,21^

#### Anxiety

The anxiety level of children aged 4 to 7 years was assessed by the visual analogue scale (VAS) for anxiety. It is a 10-cm horizontal line marked with the words ”not worried” (low score) at one end and ”very worried” (high score) at the other, with different facial expressions drawn along the line. This scale has been shown to be reliable and valid for measuring the subjective feelings of anxiety in children.^35,36^

The anxiety level of children aged 8 to 12 years was assessed by the short form of the Chinese version of the State Anxiety Scale for Children (CSAS-C).^37,38^ It is a 3-point Likert scale with scores ranging from 10 to 30. Higher scores indicate greater anxiety levels.^37,38^ It has been previously used to assess the anxiety level of Chinese children undergoing medical procedures.^36^ The Cronbach α of this scale in this current study was 0.89.

#### Additional Measures

Patient heart rate was measured with an automatic heart-rate monitoring machine to assess patients’ physiological responses to pain and anxiety.^22,23^ Children’s stress level was assessed by salivary cortisol assay.^39^ The trained RA collected patients’ saliva samples and tested them according to the manufacturer’s instructions. The length of procedure, from disinfection to band aid applied to the venipuncture site, was measured using a standard stopwatch. Health care professional satisfaction with the procedure was examined by the Staff Satisfaction Scale.^40^ It comprises 8 items, each rated by a 5-point scale ranging from 1 (strongly disagree) to 5 (strongly agree). It has been translated and used in a previous study.^38^ The Cronbach α of this scale in the current study was 0.88.

### Data Collection Procedure

A pediatric nurse in the unit screened patients’ eligibility. Those who met the inclusion criteria for recruitment and their accompanying parents were referred to the RA, who explained the study, obtained written informed consent from consenting participants, and collected baseline data.

Participants were assessed 10 minutes before (T0), during (T1), immediately after (T2), and 30 minutes after (T3) the procedure. At T0, baseline data including saliva cortisol, FPS-R score, VAS for anxiety score, CSAS-C score, and heart rate were collected. At T1, heart rate data were collected. At T2, FPS-R score, VAS for anxiety score, CSAS-C score, and heart rate were obtained from patients as well as the length of the procedure and health care professional satisfaction. At T3, saliva cortisol, FPS-R score, VAS for anxiety score, CSAS-C score, and heart rate were obtained from patients again.

### Statistical Analysis

SPSS statistical software version 24 (IBM Corp) was used for data analysis. Continuous and categorical variables were summarized by means (SD) or frequencies (percentages), as appropriate. The intention-to-treat principle was adopted for outcome comparisons between the 2 groups. The independent *t* test (or Mann-Whitney test for highly skewed data) was used as appropriate to assess the outcomes (such as length of procedure and staff satisfaction level) between the 2 groups. The generalized estimating equation (GEE) model was used to compare each of the outcome measures across the time points between the 2 groups. Hedges *g* effect size was used to estimate the effects of IVR intervention on the outcome variables. All statistical analyses were 2-sided, and the level of significance was set at *P* < .05.

## Results

A total of 155 eligible pediatric patients and their parents were approached; of these, 149 patients were randomized, with the mean age (SD) of 7.21 (2.45) years. More than half were aged between 4 and 7 years (88 [59.1%]) and female (86 [57.7%]), and nearly half were diagnosed with fever (66 [44.3%]) (Figure; Table 1). Of the randomized patients, 75 (mean [SD] age, 7.21 [2.43] years) were allocated to the intervention group and 74 (mean [SD] age, 7.21 [2.49] years) to the control group. Patients in the IVR group did not report any adverse effects such as dizziness, nausea, headache, or eye strain during and after the procedures.

**Figure.**
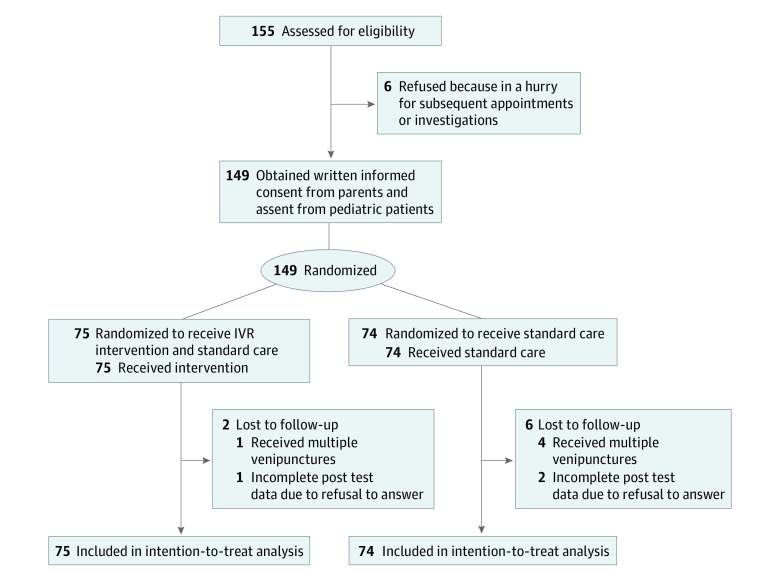
Study Flow Diagram IVR indicates immersive virtual reality.

**Table 1.** Sociodemographic and Clinical Characteristics of the Participants

Characteristic	Participants, No. (%)		
	Total (N = 149)	Intervention (n = 75)	Control (n = 74)
Age, y			
Mean (SD)	7.21 (2.45)	7.21 (2.43)	7.21 (2.49)
4-7	88 (59.1)	43 (57.3)	45 (60.8)
8-12	61 (40.9)	32 (42.7)	29 (39.2)
Sex			
Female	86 (57.7)	40 (53.3)	46 (62.2)
Male	63 (42.3)	35 (46.7)	28 (37.8)
Diagnosis			
Fever	66 (44.3)	37 (49.3)	29 (39.2)
Respiratory-related problem	21 (14.1)	8 (10.7)	13 (17.5)
Gastrointestinal problem	14 (9.4)	5 (6.7)	9 (12.2)
Orthopedic problem	12 (8.0)	6 (8.0)	6 (8.1)
Others	36 (24.2)	19 (25.3)	17 (23.0)
Hospital stays in past y, No.			
0	42 (28.2)	21 (28.0)	21 (28.4)
1-2	87 (58.4)	47 (62.7)	40 (54.0)
≥3	20 (13.4)	7 (9.3)	13 (17.6)
Previous venipuncture, No.			
0	43 (28.9)	26 (34.7)	17 (23.0)
1	57 (38.3)	30 (40.0)	27 (36.5)
2-5	39 (26.3)	15 (20.0)	24 (32.4)
≥6	10 (6.5)	4 (5.3)	6 (8.1)
Received surgery			
Yes	6 (4.0)	3 (4.0)	3 (4.1)
No	143 (96.0)	72 (96.0)	71 (95.9)
Use of analgesic medication			
Yes	32 (21.5)	15 (20.0)	17 (23.0)
No	117 (78.5)	60 (80.0)	57 (77.0)

### Pain Level

Table 2 summarizes the primary and secondary outcomes across the study points and the effect sizes. A large effect size was observed at T2 on pain scores (*d* = 0.71; 95% CI, 0.37 to 1.04). Likewise, GEE results showed that patients in the IVR group had a significantly smaller increase in pain score at T2 vs T0 than those in the control group (β = −0.78; 95% CI, −1.21 to −0.35; *P* < .001) (Table 3). Similar GEE results were observed in the subgroup analysis for patients aged 4 to 7 years (β = −1.05; 95% CI, −1.62 to −0.49; *P* < .001) with a large effect size (*d* = 0.98; 95% CI, 0.53 to 1.42) (Table 3 and Table 4).

**Table 2.** Outcome Measures Across Time Between the Control and Intervention Group Among All Children

Outcomes	Mean (SD)		
	Control (n = 74)	Intervention (n = 75)	Effect size (95% CI)
FPS-R pain score (range: 0-10)			
T0	1.09 (1.96)	0.96 (1.96)	NA
T2	4.99 (3.95)	2.24 (2.81)	0.71 (0.37 to 1.04)^a^
T3	1.57 (3.06)	0.63 (1.32)	0.25 (−0.07 to 0.57)^a^
Anxiety *z* scores			
T0	0.10 (1.02)	−0.10 (0.97)	NA
T2	0.00 (1.21)	−0.62 (0.79)	0.36 (0.03 to 0.68)^a^
T3	−0.67 (.89)	−0.91 (0.69)	0.04 (−0.29 to 0.36)^a^
Heart rate/min			
T0	101.2 (17.7)	102.3 (16.7)	NA
T1	111.2 (24.9)	108.9 (27.3)	0.23 (−0.10 to 0.56)^a^
T2	103.7 (21.4)	102.4 (26.1)	0.29 (−0.04 to 0.62)^a^
T3	97.6 (18.8)	97.7 (18.2)	0.06 (−0.27 to 0.38)^a^
Salivary cortisol			
T0	0.06 (0.04)	0.06 (0.04)	NA
T3	0.05 (0.03)	0.05 (0.03)	0.02 (−0.45 to 0.50)^a^
Length of procedure, min	6.56 (7.39)	4.43 (3.47)	0.37 (0.04 to 0.69)
Staff satisfaction	32.9 (4.0)	34.5 (4.5)	0.36 (0.04 to 0.68)

Abbreviations: FPS-R, Faces Pain Scale–Revised; NA, not applicable; T0, 10 minutes before venipuncture; T1, during venipuncture; T2, after venipuncture; T3, 30 minutes after venipuncture.

^a^ Hedges *g* effect size, which corresponds to the standardized mean difference of the mean changes at the underlying time with respect to T0 between the intervention and control groups.

**Table 3.** Generalized Estimating Equation Models for the Comparison of Each Repeated Outcome Across Time Between the Control and Intervention Groups^a^

Outcomes	Among all children		Children aged 4-7 y		Children aged 8-12 y	
	β (95% CI)	*P* value	β (95% CI)	*P* value	β (95% CI)	*P* value
**FPS-R pain score^b^**						
Group	−0.07 (−0.34 to 0.20)	.62	0.01 (−0.33 to 0.36)	.94	−0.20 (−0.64 to 0.25)	.39
T2	1.33 (1.00 to 1.66)	<.001	1.52 (1.11 to 1.93)	<.001	1.05 (0.51 to 1.59)	<.001
T3	0.06 (−0.27 to 0.39)	.72	0.11 (−0.29 to 0.51)	.59	−0.02 (−0.59 to 0.56)	.95
Group × T2	−0.78 (−1.21 to −0.35)	<.001	−1.05 (−1.62 to −0.49)	<.001	−0.38 (−1.04 to 0.29)	.27
Group × T3	−0.21 (−0.63 to 0.21)	.33	−0.15 (−0.69 to 0.39)	.58	−0.28 (−0.95 to 0.40)	.43
**Anxiety *z* scores**						
Group	−0.21 (−0.52 to 0.11)	.21	−0.31 (−1.72 to 1.10)	.67	−1.89 (−4.38 to 0.61)	.14
T2	−0.11 (−0.40 to 0.18)	.47	0.72 (−0.49 to 1.92)	.24	−3.00 (−5.17 to −0.83)	.007
T3	−0.77 (−1.06 to −0.48)	<.001	−2.38 (−3.67 to −1.09)	<.001	−4.45 (−6.61 to −2.29)	<.001
Group × T2	−0.41 (−0.76 to −0.05)	.03	−2.28 (−3.88 to −0.67)	.006	0.03 (−2.46 to 2.51)	.98
Group × T3	−0.04 (−0.40 to 0.32)	.83	−0.06 (−1.71 to 1.58)	.94	−0.27 (−3.00 to 2.46)	.85
**Heart rate/min**						
Group	2.13 (−3.71 to 7.96)	.48	2.76 (−4.80 to 10.32)	.47	1.87 (−6.10 to 9.84)	.65
T1	10.01 (4.82 to 15.20)	<.001	12.93 (5.16 to 20.70)	.001	5.59 (0.35 to 10.82)	.04
T2	2.49 (−1.96 to 6.94)	.27	4.25 (−2.27 to 10.77)	.20	−0.31 (−5.44 to 4.82)	.91
T3	−3.61 (−7.99 to 0.78)	.11	−5.26 (−11.78 to 1.25)	.11	−1.10 (−5.76 to 3.55)	.64
Group × T1	−4.05 (−10.90 to 2.81)	.25	−6.81 (−17.22 to 3.60)	.20	0.41 (−7.17 to 8.00)	.92
Group × T2	−3.61 (−9.47 to 2.24)	.23	−2.89 (−11.43 to 5.67)	.51	−3.99 (−11.28 to 3.30)	.28
Group × T3	−2.12 (−8.20 to 3.96)	.50	−1.08 (−9.60 to 7.44)	.80	−3.57 (−11.39 to 4.24)	.37
**Salivary cortisol**						
Group	−0.002 (−0.019 to 0.015)	.83	−0.004 (−0.027 to 0.019)	.75	0.001 (−0.023 to 0.025)	.94
T3	−0.009 (−0.023 to 0.004)	.18	−0.011 (−0.031 to 0.008)	.24	−0.006 (−0.024 to 0.012)	.52
Group × T3	−0.003 (−0.022 to 0.017)	.78	−0.002 (−0.029 to 0.025)	.90	−0.004 (−0.031 to 0.023)	.76

Abbreviations: FPS-R, Faces Pain Scale–Revised; T1, during venipuncture; T2, after venipuncture; T3, 30 minutes after venipuncture.

^a^ Only the model estimates of regression coefficients (β) of the dummy variables for the group (group 0, control [reference]; group 1, intervention), time points (T1, T2, and T3, with the measurement taken 10 minutes before venipuncture as reference), and time points and group interaction terms (group × T1, group × T2, and group × T3) are shown for the generalized estimating equation models.

^b^ FPS-R pain score was square root transformed before being entered into the GEE analysis.

**Table 4.** Outcome Measures Across Time Between the Control and Intervention Group Stratified by Age Group

Outcomes	Control	Intervention	Effect size (95% CI)^a^
**Children aged 4-7 y**			
No.	45	43	NA
FPS-R pain score (range: 0-10)			
T0	0.89 (1.84)	0.98 (2.06)	NA
T2	5.50 (4.09)	2.14 (2.88)	0.98 (0.53 to 1.42)
T3	1.47 (3.00)	0.84 (1.54)	0.22 (−0.20 to 0.64)
VAS for anxiety			
T0	4.42 (3.39)	4.12 (3.43)	NA
T2	5.14 (4.07)	2.56 (2.92)	0.60 (0.17 to 1.02)
T3	2.04 (2.64)	1.67 (2.37)	0.02 (−0.40 to 0.43)
Heart rate/min			
T0	105.5 (17.1)	106.7 (16.5)	NA
T1	118.4 (23.2)	113.9 (31.0)	0.35 (−0.08 to 0.78)
T2	109.7 (19.1)	109.6 (28.8)	0.31 (−0.12 to 0.74)
T3	100.2 (19.8)	101.8 (19.8)	−0.04 (−0.47 to 0.38)
Salivary cortisol			
T0	0.06 (0.04)	0.06 (0.03)	NA
T3	0.05 (0.03)	0.04 (0.03)	0.06 (−0.61 to 0.74)
Length of procedure, min	6.98 (7.00)	5.20 (3.67)	0.31 (−0.11 to 0.73)
Staff satisfaction	32.1 (3.9)	33.0 (4.4)	0.21 (−0.21 to 0.63)
**Children aged 8-12 y**			
No.	29	32	NA
FPS-R pain score (range: 0-10)			
T0	1.41 (2.13)	0.94 (1.83)	NA
T2	4.21 (3.64)	2.39 (2.75)	0.31 (−0.20 to 0.82)
T3	1.72 (3.19)	0.34 (0.90)	0.27 (−0.23 to 0.78)
State Anxiety scale			
T0	19.4 (5.3)	17.6 (4.7)	NA
T2	16.4 (5.7)	14.5 (3.4)	−0.05 (−0.56 to 0.45)
T3	15.0 (5.4)	12.8 (3.2)	0.05 (−0.45 to 0.55)
Heart rate/min			
T0	94.7 (16.7)	96.6 (15.4)	NA
T1	100.3 (23.7)	102.6 (20.5)	−0.03 (−0.53 to 0.48)
T2	94.4 (21.9)	92.6 (18.4)	0.24 (−0.27 to 0.74)
T3	93.6 (16.8)	91.9 (14.0)	0.24 (−0.26 to 0.75)
Salivary cortisol			
T0	0.05 (0.03)	0.06 (0.04)	NA
T3	0.05 (0.03)	0.05 (0.03)	−0.02 (−0.70 to 0.66)
Length of procedure, min	5.90 (8.03)	3.38 (2.92)	0.42 (−0.09 to 0.93)
Staff satisfaction	34.2 (3.9)	36.4 (3.9)	0.57 (0.06 to 1.09)

Abbreviations: FPS-R, Faces Pain Scale–Revised; NA, not applicable; T0, 10 minutes before venipuncture; T1, during venipuncture; T2, after venipuncture; T3, 30 minutes after venipuncture; VAS, visual analog scale.

^a^ Hedges *g* effect size, which corresponds to the standardized mean difference between the intervention and control group, or the mean changes at the underlying time point with respect to T0 between the 2 groups.

### Anxiety

A small effect size on anxiety scores was found at T2 (*d* = 0.36; 95% CI, 0.03 to 0.68) (Table 2). According to the GEE results, the IVR group showed a significantly greater reduction in anxiety scores at T2 vs T0 compared with the control group (β = −0.41; 95% CI, −0.76 to −0.05; *P* = .03) (Table 3). The positive effect on anxiety score at T2 was also observed in the subgroup analysis for patients aged 4 to 7 years (β = −2.28; 95% CI, −3.88 to −0.67; *P* = .006), with a moderate effect size (*d* = 0.60; 95% CI, 0.17 to 1.02) (Tables 3 and 4).

### Heart Rate and Stress

The intervention group showed smaller increase in heart rate at T1 and T2 with respect to T0 and a greater reduction in salivary cortisol at T3 with respect to T0 compared with the control group. However, statistical significance was not reached (Table 3).

### Length of Procedure

Independent *t* test showed that the length of procedure in the IVR group (mean [SD] duration, 4.43 [3.47] minutes) was significantly shorter than that in the control group (mean [SD] duration, 6.56 [7.39] minutes; *P* = .03). Subgroup analysis by age group suggests similar results, but statistical significance was not reached (Table 4).

### Staff Satisfaction

Independent *t* test revealed that staff satisfaction score in the intervention group (mean [SD] score, 34.5 [4.5]) was significantly higher than that in the control group (mean [SD] score, 32.9 [4.0); *P* = .03). Subgroup analysis suggested similar results among in the older age group only (Table 4).

## Discussion

This randomized clinical trial found that an IVR intervention incorporating distraction and procedural information, rather than standard care alone, substantially reduced self-reported pain, self-reported anxiety, and length of procedure among pediatric patients undergoing venipuncture. Additionally, a statistically significantly higher level of staff satisfaction was noted in the IVR group than in the control group. Although the IVR group showed a smaller increase in heart rate and a greater reduction in salivary cortisol than the control group after the procedure, statistical significance was not observed.

This trial extended previous studies and, to our knowledge, was the first randomized clinical trial to distract pediatric patients while simultaneously offering procedural information during venipuncture using IVR. Overall results showed that the IVR intervention effectively mitigated pain and anxiety in children undergoing venipuncture, which is concordant with previous studies that provided distraction using VR.^41^ Subgroup analysis revealed a large effect on pain and a moderate effect on anxiety immediately after venipuncture in the younger age group only. This echoed a systematic review suggesting that VR interventions may have better effects on younger children than older children.^41^

Previous studies have used IVR across a broad age range and various needle-related procedures, of which only a few studies have tailored contents according to medical procedures and the developmental level of the children.^13,14,42^ Nevertheless, the limited evidence on VR equivocally suggests that tailored content is more effective than ready-made content in mitigating pain and distress.^17,32,43^ As such, the positive results of our intervention may be attributed to the tailored and synchronized VR scenarios. Specifically, our VR scenarios not only provided age-appropriate procedural information, but the contents were synchronized with real-time procedures so that children could gain a sense of control and be prepared when the needle was inserted.

This trial used an objective measure of salivary cortisol to assess children’s stress levels before and 30 minutes after venipuncture. However, we did not find significant between-group differences in the changes of salivary cortisol. Similarly, a previous study comparing the effects of pharmacological (jet lidocaine) and nonpharmacological interventions (the Buzzy, bubble-blowing, and inhalation aromatherapy) in children undergoing phlebotomy found no between-group difference in salivary cortisol levels collected 25 minutes after the procedures.^44^ Given the short duration of the venipuncture, it was possible that the cortisol level was only raised for a short time and had already returned to normal 30 minutes after venipuncture.^45^ Future studies using other objective measures to assess children’s stress level are warranted.

The current study found that IVR intervention shortened the length of procedures and improved staff satisfaction with the procedures. A probable explanation is that patients were fully engaged in IVR and demonstrated higher procedural compliance and cooperativeness, which led to reduced procedure duration and increased health care professional satisfaction. Similar findings were noted in previous studies showing that VR decreased the need for restraining pediatric patients during needle-related procedures and increased health care professional satisfaction.^6,11,46^

### Implications

Given that venipuncture is one of the most frequently performed and distressing procedures in hospitalized pediatric patients, the results generated in this study may contribute tangible improvements to patient care and outcomes. Our encouraging results imply that an IVR intervention can be adopted as a high-quality clinical intervention for simultaneously offering distraction and procedural information to mitigate pain and anxiety in children undergoing venipuncture. In contrast to previously used approaches (eg, playing with toys and soothing by adults) that are time and resource-intensive,^13^ IVR can be used virtually by numerous patients simultaneously at any time and place. Compared with computer-based VR, which is bulky, the use of smartphone-based VR in our study is more practical for clinical implementation.^47^ Furthermore, the principles of this intervention can be generalized and extended to other needle-related or pain- and anxiety-inducing medical procedures beyond venipuncture.

As IVR devices are becoming increasingly affordable, health care professionals may benefit from integrating IVR interventions into routine practices. However, prior to clinical implementation, measures need to be considered to ensure that strict infection control is maintained in hospital settings, especially during the COVID-19 pandemic, and that staff are trained to supervise interventions and operate IVR equipment.

### Limitations

This study has several limitations. First, due to the nature of the IVR intervention, participants were not blinded to the study. Nevertheless, children are not expected to change their behavior even if they are aware of their participation in an intervention.^48,49,50^ Second, the results may not be generalized to younger patients in other settings, such as outpatient clinics. Third, this study was conducted at a single site; future studies should consider recruiting participants at multiple sites with larger and more diverse samples to increase the generalizability of the findings and the validity of the evidence generated.

## Conclusions

In this randomized clinical trial of an IVR intervention for pediatric patients undergoing venipuncture, pain, anxiety, length of procedure, and staff satisfaction with the procedures were significantly improved after the IVR intervention. The results also suggested that both distraction and procedural information can be offered to children during an IVR intervention. Given that IVR is becoming more affordable and accessible, it could be used to improve children’s experiences of needle-related or pain- and anxiety-inducing medical procedures.

## Notes

Supplement 1.
Trial Protocol

Supplement 2. **eFigure.** Screenshots of the Scenarios

Supplement 3.
Data Sharing Statement

## References

[1] Walther-Larsen S, Pedersen MT, Friis SM, . Pain prevalence in hospitalized children: a prospective cross-sectional survey in four Danish university hospitals. Acta Anaesthesiol Scand. 2017;61(3):328-337. doi:10.1111/aas.12846 28032329

[2] Stevens BJ, Abbott LK, Yamada J, ; CIHR Team in Children’s Pain. Epidemiology and management of painful procedures in children in Canadian hospitals. CMAJ. 2011;183(7):E403-E410. doi:10.1503/cmaj.101341 21464171 PMC3080557

[3] Pate JT, Blount RL, Cohen LL, Smith AJ. Childhood medical experience and temperament as predictors of adult, functioning in medical situations. Child Health Care. 1996;25(4):281-298. doi:10.1207/s15326888chc2504_4

[4] Humphrey GB, Boon CM, van Linden van den Heuvell GF, van de Wiel HB. The occurrence of high levels of acute behavioral distress in children and adolescents undergoing routine venipunctures. Pediatrics. 1992;90(1 Pt 1):87-91. doi:10.1542/peds.90.1.87 1614786

[5] Vest E, Armstrong M, Olbrecht VA, . Association of pre-procedural anxiety with procedure-related pain during outpatient pediatric burn care: a pilot study. J Burn Care Res. Published online August 1, 2022. doi:10.1093/jbcr/irac108 35913793

[6] Kennedy RM, Luhmann J, Zempsky WT. Clinical implications of unmanaged needle-insertion pain and distress in children. Pediatrics. 2008;122(suppl 3):S130-S133. doi:10.1542/peds.2008-1055e 18978006

[7] Noel M, McMurtry CM, Chambers CT, McGrath PJ. Children’s memory for painful procedures: the relationship of pain intensity, anxiety, and adult behaviors to subsequent recall. J Pediatr Psychol. 2010;35(6):626-636. doi:10.1093/jpepsy/jsp096 19889718

[8] Karlsson K, Rydström I, Nyström M, Enskär K, Dalheim Englund AC. Consequences of needle-related medical procedures: a hermeneutic study with young children (3-7 years). J Pediatr Nurs. 2016;31(2):e109-e118. doi:10.1016/j.pedn.2015.09.008 26603292

[9] McMurtry CM, Pillai Riddell R, Taddio A, ; HELPinKids&Adults Team. Far from “just a poke”: common painful needle procedures and the development of needle pain. Clin J Pain. 2015;31(10)(suppl):S3-S11. doi:10.1097/AJP.0000000000000272 26352920 PMC4900413

[10] Smith RW, Shah V, Goldman RD, Taddio A. Caregivers’ responses to pain in their children in the emergency department. Arch Pediatr Adolesc Med. 2007;161(6):578-582. doi:10.1001/archpedi.161.6.578 17548763

[11] Cohen LL, Blount RL, Panopoulos G. Nurse coaching and cartoon distraction: an effective and practical intervention to reduce child, parent, and nurse distress during immunizations. J Pediatr Psychol. 1997;22(3):355-370. doi:10.1093/jpepsy/22.3.355 9212553

[12] Czarnecki ML, Turner HN, Collins PM, Doellman D, Wrona S, Reynolds J. Procedural pain management: a position statement with clinical practice recommendations. Pain Manag Nurs. 2011;12(2):95-111. doi:10.1016/j.pmn.2011.02.003 21620311

[13] Birnie KA, Noel M, Parker JA, . Systematic review and meta-analysis of distraction and hypnosis for needle-related pain and distress in children and adolescents. J Pediatr Psychol. 2014;39(8):783-808. doi:10.1093/jpepsy/jsu029 24891439 PMC4138805

[14] Birnie KA, Noel M, Chambers CT, Uman LS, Parker JA. Psychological interventions for needle-related procedural pain and distress in children and adolescents. Cochrane Database Syst Rev. 2018;10(10):CD005179. doi:10.1002/14651858.CD005179.pub4 30284240 PMC6517234

[15] Uman LS, Birnie KA, Noel M, . Psychological interventions for needle-related procedural pain and distress in children and adolescents. Cochrane Database Syst Rev. 2013;10(10):CD005179. doi:10.1002/14651858.CD005179.pub3 24108531

[16] Dahlquist LM, Busby SM, Slifer KJ, . Distraction for children of different ages who undergo repeated needle sticks. J Pediatr Oncol Nurs. 2002;19(1):22-34. doi:10.1053/jpon.2002.30009 11813138

[17] Kuo HC, Pan HH, Creedy DK, Tsao Y. Distraction-based interventions for children undergoing venipuncture procedures: a randomized controlled study. Clin Nurs Res. 2018;27(4):467-482. doi:10.1177/1054773816686262 28038497

[18] Brown NJ, Kimble RM, Rodger S, Ware RS, Cuttle L. Play and heal: randomized controlled trial of Ditto intervention efficacy on improving re-epithelialization in pediatric burns. Burns. 2014;40(2):204-213. doi:10.1016/j.burns.2013.11.024 24360745

[19] Won AS, Bailey J, Bailenson J, Tataru C, Yoon IA, Golianu B. Immersive virtual reality for pediatric pain. Children (Basel). 2017;4(7):E52. doi:10.3390/children4070052 28644422 PMC5532544

[20] Chirico A, Lucidi F, De Laurentiis M, Milanese C, Napoli A, Giordano A. Virtual reality in health system: beyond entertainment—a mini-review on the efficacy of VR during cancer treatment. J Cell Physiol. 2016;231(2):275-287. doi:10.1002/jcp.25117 26238976

[21] Gold JI, Kim SH, Kant AJ, Joseph MH, Rizzo AS. Effectiveness of virtual reality for pediatric pain distraction during I.V. placement. Cyberpsychol Behav. 2006;9(2):207-212. doi:10.1089/cpb.2006.9.207 16640481

[22] Gershon J, Zimand E, Pickering M, Rothbaum BO, Hodges L. A pilot and feasibility study of virtual reality as a distraction for children with cancer. J Am Acad Child Adolesc Psychiatry. 2004;43(10):1243-1249. doi:10.1097/01.chi.0000135621.23145.05 15381891

[23] Gold JI, SooHoo M, Laikin AM, Lane AS, Klein MJ. Effect of an immersive virtual reality intervention on pain and anxiety associated with peripheral intravenous catheter placement in the pediatric setting: a randomized clinical trial. JAMA Netw Open. 2021;4(8):e2122569. doi:10.1001/jamanetworkopen.2021.22569 34432011 PMC8387848

[24] Windich-Biermeier A, Sjoberg I, Dale JC, Eshelman D, Guzzetta CE. Effects of distraction on pain, fear, and distress during venous port access and venipuncture in children and adolescents with cancer. J Pediatr Oncol Nurs. 2007;24(1):8-19. doi:10.1177/1043454206296018 17185397

[25] Melzack R, Wall PD. Pain mechanisms: a new theory. Science. 1965;150(3699):971-979. doi:10.1126/science.150.3699.971 5320816

[26] Lazarus RS, Folkman S. Stress, Appraisal and Coping.Springer; 1984.

[27] Wong CL, Lui MMW, Choi KC. Effects of immersive virtual reality intervention on pain and anxiety among pediatric patients undergoing venipuncture: a study protocol for a randomized controlled trial. Trials. 2019;20(1):369. doi:10.1186/s13063-019-3443-z 31221208 PMC6585051

[28] De More M, Cohen LL. Distraction for pediatric immunization pain: a critical review. J Clin Psychol Med Settings. 2005;12(4):281-291. doi:10.1007/s10880-005-7813-1

[29] Jaaniste T, Brett H, Von Baeyer CL. Providing children with information about forthcoming medical procedures: a review and synthesis. Clin Psychol Sci Pract. 2007;14(2):124-143. doi:10.1111/j.1468-2850.2007.00072.x

[30] Piaget J. The Origins of Intelligence in Children. Norton; 1963.

[31] Blankenburg M, Boekens H, Hechler T, . Reference values for quantitative sensory testing in children and adolescents: developmental and gender differences of somatosensory perception. Pain. 2010;149(1):76-88. doi:10.1016/j.pain.2010.01.011 20138430

[32] Hoffman HG, Richards TL, Van Oostrom T, . The analgesic effects of opioids and immersive virtual reality distraction: evidence from subjective and functional brain imaging assessments. Anesth Analg. 2007;105(6):1776-1783. doi:10.1213/01.ane.0000270205.45146.db 18042882

[33] Wright JA. Animation Writing and Development: From Script Development to Pitch. 2nd ed. Focal Press; 2013.

[34] Hicks CL, von Baeyer CL, Spafford PA, van Korlaar I, Goodenough B. The Faces Pain Scale–Revised: toward a common metric in pediatric pain measurement. Pain. 2001;93(2):173-183. doi:10.1016/S0304-3959(01)00314-1 11427329

[35] Bringuier S, Dadure C, Raux O, Dubois A, Picot MC, Capdevila X. The perioperative validity of the visual analog anxiety scale in children: a discriminant and useful instrument in routine clinical practice to optimize postoperative pain management. Anesth Analg. 2009;109(3):737-744. doi:10.1213/ane.0b013e3181af00e4 19690240

[36] Wong CL, Ip WY, Kwok BMC, Choi KC, Ng BKW, Chan CWH. Effects of therapeutic play on children undergoing cast-removal procedures: a randomised controlled trial. BMJ Open. 2018;8(7):e021071. doi:10.1136/bmjopen-2017-021071 29980545 PMC6042539

[37] Li HC, Lopez V. Development and validation of a short form of the Chinese version of the State Anxiety Scale for Children. Int J Nurs Stud. 2007;44(4):566-573. doi:10.1016/j.ijnurstu.2005.12.004 16464452

[38] Li HC, Wong ML, Lopez V. Factorial structure of the Chinese version of the State Anxiety Scale for Children (short form). J Clin Nurs. 2008;17(13):1762-1770. doi:10.1111/j.1365-2702.2008.02284.x 18578780

[39] Patil SJ, Shah PP, Patil JA, Shigli A, Patil AT, Tamagond SB. Assessment of the changes in the stress-related salivary cortisol levels to the various dental procedures in children. J Indian Soc Pedod Prev Dent. 2015;33(2):94-99. doi:10.4103/0970-4388.155116 25872625

[40] Tyson ME, Bohl DD, Blickman JG. A randomized controlled trial: child life services in pediatric imaging. Pediatr Radiol. 2014;44(11):1426-1432. doi:10.1007/s00247-014-3005-1 24801818

[41] Eijlers R, Utens EMWJ, Staals LM, . Systematic review and meta-analysis of virtual reality in pediatrics: effects on pain and anxiety. Anesth Analg. 2019;129(5):1344-1353. doi:10.1213/ANE.0000000000004165 31136330 PMC6791566

[42] Wang Y, Guo L, Xiong X. Effects of virtual reality-based distraction of pain, fear, and anxiety during needle-related procedures in children and adolescents. Front Psychol. 2022;13:842847. doi:10.3389/fpsyg.2022.842847 35519646 PMC9063726

[43] Miller K, Rodger S, Bucolo S, Greer R, Kimble RM. Multi-modal distraction: using technology to combat pain in young children with burn injuries. Burns. 2010;36(5):647-658. doi:10.1016/j.burns.2009.06.199 19889502

[44] Küçük Alemdar D, Yaman Aktaş Y. The use of the buzzy, jet lidokaine, bubble-blowing and aromatherapy for reducing pediatric pain, stress and fear associated with phlebotomy. J Pediatr Nurs. 2019;45:e64-e72. doi:10.1016/j.pedn.2019.01.010 30711327

[45] Hanrahan K, McCarthy AM, Kleiber C, Lutgendorf S, Tsalikian E. Strategies for salivary cortisol collection and analysis in research with children. Appl Nurs Res. 2006;19(2):95-101. doi:10.1016/j.apnr.2006.02.001 16728293

[46] Chan E, Hovenden M, Ramage E, . Virtual reality for pediatric needle procedural pain: two randomized clinical trials. J Pediatr. 2019;209:160-167.e4. doi:10.1016/j.jpeds.2019.02.034 31047650

[47] Xiang H, Shen J, Wheeler KK, . Efficacy of smartphone active and passive virtual reality distraction vs standard care on burn pain among pediatric patients: a randomized clinical trial. JAMA Netw Open. 2021;4(6):e2112082. doi:10.1001/jamanetworkopen.2021.12082 34152420 PMC8218073

[48] Wong CL, Li CK, Chan CWH, . Virtual reality intervention targeting pain and anxiety among pediatric cancer patients undergoing peripheral intravenous cannulation: a randomized controlled trial. Cancer Nurs. 2021;44(6):435-442. doi:10.1097/NCC.0000000000000844 32511154

[49] Wong CL, Li CK, Choi KC, . Effects of immersive virtual reality for preventing and managing anxiety, nausea and vomiting among paediatric cancer patients receiving their first chemotherapy: a study protocol for an exploratory trial. PLoS One. 2021;16(10):e0258514. doi:10.1371/journal.pone.0258514 34648568 PMC8516310

[50] Lee Wong C, Li CK, Choi KC, . Effects of immersive virtual reality for managing anxiety, nausea and vomiting among paediatric cancer patients receiving their first chemotherapy: an exploratory randomised controlled trial. Eur J Oncol Nurs. 2022;61:102233. doi:10.1016/j.ejon.2022.102233 36401916

