# Supplementary material for: Effects of an Immersive Virtual Reality Intervention on Pain and Anxiety Among Pediatric Patients Undergoing Venipuncture: A Randomized Clinical Trial
Source: JAMA Netw Open. 2023 Feb 16;6(2):e230001. doi: 10.1001/jamanetworkopen.2023.0001 (PMC9936341; doi:10.1001/jamanetworkopen.2023.0001)
Supplement: Supplement 1. — Trial Protocol [file jamanetwopen-e230001-s001.pdf]

Effects of immersive virtual reality intervention on pain and anxiety among pediatric patients undergoing venipuncture: A randomized controlled trial

Lead PI: *Dr. Wong Cho Lee, Assistant Professor, the Nethersole School of Nursing, Faculty of Medicine, the Chinese University of Hong Kong*

Site PI: *Ms Lui Mei Wa, Ward Manager, Department of Paediatrics and Adolescent Medicine, Tseung Kwan O Hospital*

Abstract

Venipuncture is one of the most painful and distressing procedure experienced by pediatric patients. Unmanaged pain has short- and long-term consequences to patients. Moreover, it prolongs the procedure and consumes additional manpower while decreasing the satisfaction of health-care providers. Evidence suggests that distraction combined with age-appropriate procedural information can effectively decrease procedural pain and anxiety in pediatric patients. This integrated approach, however, may be difficult to implement and consumes high amounts of manpower and time.

Immersive virtual reality (IVR) has become possible with technological advancement. It can simultaneously provide complete distraction and procedural information to patients. However, its effects on pediatric patients undergoing venipuncture are unknown.

Guided by the Gate Control Theory and the Lazarus and Folkman's Theory, this study aims to examine the effects of IVR intervention on reducing the pain, anxiety and stress experienced by pediatric patients undergoing venipuncture. It will also examine the effects of IVR on the duration of venipuncture and the satisfaction of health-care providers for the procedure. Its cost-effectiveness will also be evaluated. A randomized controlled trial with repeated assessments will be conducted. A total of 250 pediatric patients aged 4–12 years will

be recruited from a regional public hospital and randomly assigned to either the intervention or control group. The study will use two age-appropriate IVR modules that consist of procedural information developed by the lead principal investigator. The intervention group will receive IVR intervention, whereas the control group will receive standard care only. 10 health-care providers involved in the venipuncture procedure will also be invited to participate to assess their satisfaction level towards the procedure. The primary outcome is child-reported pain. Secondary outcomes include child-reported anxiety, heart rate, salivary cortisol, length of procedure, and satisfaction of health care providers with respect to the venipuncture procedure. The cost-effectiveness of IVR intervention will be compared with that of standard care. Outcome evaluation will be conducted at four time points: 10 minutes before, during, and immediately after and 30 minutes after the procedure. Intention to treat and generalized estimating equation model will be used to analyze the data.

This study is the first of its kind to adopt IVR intervention with age-appropriate procedural information for pediatric patients undergoing venipuncture. Findings of the proposed study may (1) provide a novel, facile, and cost-effective intervention that can be used virtually at any time and place to manage pain and anxiety; and (2) shed light on the global trends of research and clinical development of IVR as an intervention for other painful and stressful medical procedures.

Background of research

Hospitalized children experience pain and anxiety from invasive procedures and/or their underlying diseases [1]. Venipuncture, a frequently performed needle-related procedure, is the most frightening experience and a common source of moderate to severe pain for pediatric patients [1-2]. The pain associated with the procedure is secondary only to the illness itself [3]. Approximately 83% of children aged 2.5–6 years, 51% of children aged 7–12 years, and 28% of adolescents (>12 years of age) who underwent venipuncture reported high levels of distress during the procedure [4]. However, less than 10% of venipuncture procedures are provided with pain management [2].

Inadequate pain management can negatively affect children, parents, and medical institutions [5-10]. Unmanaged needle-related procedural pain in children is associated with increased pain and stress during subsequent procedures, fear and avoidance of medical care, or even the development of needle phobias that persist into adulthood [3, 6-8]. Failure to manage needle-related pain may increase the manpower, resources, and time required to complete procedures, consequently reducing health-care providers' satisfaction with respect to the procedures [5, 10].

Pre-procedural local analgesics have been traditionally used to manage needle-related procedural pain. However, high levels of pain and distress were reported in children even with pharmacological interventions [11-12]. Thus, clinical guidelines highlight the important role of non-pharmacological interventions alone or as clinical adjunct in managing procedural pain [13].

Non-pharmacological interventions

Various studies have suggested non-pharmacological interventions, such as distraction, procedural information provision, hypnosis, and cognitive behavioral therapy, to manage the pain and anxiety experienced by pediatric patients undergoing needle-related procedures [14-

16]. However, distraction and information provision are more feasible to implement than previously suggested interventions because many clinicians are not trained in hypnosis or cognitive behavioral therapy.

Distraction is the most effective non-pharmacological intervention for mitigating the pain and anxiety experienced by pediatric patient undergoing needle-related procedures [14-16], particularly in children aged below 12 years [16]. A review of 26 studies involving 2,548 children aged 2–19 years showed that needle-related procedures have been performed with various distractors, such as listening to music; watching cartoons; playing with toys; and mother-directed distractions, like soothing [14]. However, further research is needed to identify the most efficacious type of distractor [16]. Nevertheless, the review suggested that distractions without adult involvement (supportive role articulated for the adult within the intervention) and child choice effectively reduce the intensity of self-reported distress [14]. In addition, developmentally age-appropriate distractors for children are important for successful pain management [16-17]. Two recent studies further confirmed that distraction intervention integrated with procedural information produce significant outcomes [18-19]. However, simultaneously providing age-appropriate distraction and procedural information may be difficult and may likely increase the workload of hospital staff. Most importantly, previously employed distractors (e.g. music, cartoon, or toys) failed to provide complete distraction because children still inevitably see the needles, which is the most distress-evoking experience in needle-related procedures [5-6]. Thus, intervention that can provide age-appropriate procedural information and completely distract the attention of children from painful stimuli should be identified.

Immersive Virtual Reality

Immersive virtual reality (IVR) may help overcome the above obstacles. It provides a means of human/computer interaction, wherein a human becomes an active participant in a

virtual environment created through a head-mounted display [20]. The user is immersed and actively participates in the virtual environment as it changes in real time with the user's movements [21]. Moreover, it can be used at any time and place in clinical settings without requiring extra manpower. Thus, it can be easily provided as an intervention for patients. IVR modules can also be tailored to provide health information to the user. The cost of IVR equipment has also become increasingly affordable. For instance, the price of a commercially available disposable IVR headset (VR Goggles) is only about HK $15. Distraction-based IVR intervention has already been implemented in the Lucile Packard Children’s Hospital at Stanford in the United States [22].

Studies of the Effects of IVR on Procedural Pain and Anxiety

A large body of evidence supports the efficacy of IVR in reducing pain, anxiety, and stress among pediatric patients undergoing burn care or cancer treatments [20-21]. With regard to needle-related procedures, a randomized control trial was conducted with 20 pediatric patients aged 8–12 years and who required peripheral intravenous access [11]. The intervention group received IVR prior to the procedure, whereas the control group received local anesthetic spray without IVR. Pain was measured using the Faces Pain Scale-Revised (FPS-R). Participants in the intervention group reported that their perceived pain was not significantly different, whereas those in the control group reported a four-fold increase in pain following the procedure [11]. Two studies involved pediatric oncology patients aged between 7–19 years and who were undergoing port access (insertion of a needle into an implanted device to facilitate blood collection or injections). Participants in the intervention group were immersed in a virtual gorilla habitat. Results found that the heart rates of participants in the intervention group significantly decreased, indicating that children in the intervention group did not experience as much pain and anxiety as those in the control group [23-24]. Another study on children with cancer (aged 5–18 years) undergoing venipuncture

(*n* = 4) or port access (*n* = 46) allowed patients in the intervention group to select from various immersive and non-immersive distractors (such as books, IVR, music, and video games) [25]. The self-reported pain of patients in the IVR group was no different. However, a reduction of fear and distress was reported by the outcome assessors in IVR group.

The research team led by the lead principal investigator (lead PI) is conducting a pilot trial on the feasibility and acceptability of IVR as a distraction intervention for pediatric oncology patients undergoing needle-related procedures. Preliminary results suggested that IVR is feasible and is acceptable to children, parents, and health care providers in Hong Kong. Patients in the intervention group reported reduction in pain and anxiety score after IVR.

Although previous studies have provided some positive findings regarding the effects of IVR on needle-related procedural pain and anxiety, these studies involved small sample sizes (*n* = 20 to 59) [11, 23-25] and recruited children of various developmental ages (5–19 years) without using age-appropriate IVR modules [11, 23-25]. Most importantly, all these studies adopted IVR as a distraction intervention only. To our knowledge, no study has utilized IVR to provide distraction and procedural information to patients undergoing venipuncture. Given that venipuncture is the most painful and fearful procedure for pediatric patients [1-4], future large-scale studies on the efficacy of IVR to provide complete distraction and procedural information to pediatric patients undergoing venipuncture may offer crucial insight on the application of this approach. The satisfaction of health care providers and the cost-effectiveness of IVR intervention should also be examined to aid decisions for the application of this approach in routine clinical practice.

Theoretical Frameworks

The Gate Control Theory and Lazarus and Folkman's theory provide the theoretical underpinning of this study [26-27]. The gate control theory suggests that peripheral nerves

become excited when cells are damaged. Impulses from nerves pass along to spinal cord systems and other neuroanatomical structures before reaching the cerebral cortex where pain is perceived. The gate control system in the spinal cord opens and closes to modulate pain perception. If non-nociceptive input (IVR) exceeds the nociceptive (pain) input, then the gate can partially or entirely close, blocking the transmission of the pain signal to the brain. The theory also proposes that pain signals that descend from the brain through the gate can be amplified by emotional experiences, such as anxiety and stress. Lazarus and Folkman's theory [27] states that an individual's evaluations of anxiety and stress-provoking experiences are influenced by their perceptions of control over a potential threat. Providing information may help improve pediatric patients' sense of control over a procedure [18-19, 28].

In the context of these two theories, IVR functions by providing multisensory input to divert the children's conscious attention from venipuncture-associated pain, thus helping close gate control and decreasing pain perception [29]. The analgesic effects of IVR have been supported by the results of functional magnetic resonance imaging assessment [30]. Providing procedural information through IVR modules further help increases the children's sense of control over the procedure, thereby reducing their stress and anxiety, as well as pain signals transmitted through the gate control system. Therefore, IVR intervention will likely reduce pain, anxiety and stress; these changes are evidenced by physiological changes in the heart rates and salivary cortisol levels of the patients. Thus, patients may show increased procedural compliance, consequently decreasing the length and cost of the procedure while possibly improving the satisfaction of the health-care providers with the procedure [5, 10].

Aim

This study aims to examine the effects of IVR intervention on reducing pain, anxiety, stress, and length of procedure among pediatric patients undergoing venipuncture. The satisfaction ratings of health care providers toward the procedures and the cost effectiveness

of the IVR intervention will also be evaluated.

Hypotheses for testing:

(1) Compared with standard care, IVR intervention significantly reduces pain, anxiety, stress, and length of procedure in pediatric patients undergoing venipuncture;

(2) Compared with standard care, IVR intervention significantly improves the satisfaction of health care providers toward the venipuncture procedures;

(3) IVR intervention is significantly more cost-effective than standard care for pediatric patients undergoing venipuncture.

Research plan and methodology

Study Design

This is a two-arm parallel randomized controlled trial.

Setting and Participants

This study will be conducted in the Department of Paediatrics & Adolescent Medicine, Tseung Kwan O Hospital. The unit admit general pediatric patients. Venipuncture will be performed by a doctor or trained phlebotomist and will be conducted in the treatment room. No local analgesics will be applied before the procedure. Usually at least one staff (nurse or health care assistants) will assist in the procedure (by saying comfort words and restraining the child from vigorous movement). Parents will wait outside the treatment room during the procedures.

Eligible pediatric patients: (1) aged between 4 and 12 years; (2) scheduled to undergo venipuncture; and (3) able to communicate in Cantonese and follow instructions. Potential participants will be excluded according to the following: (1) identified cognitive and learning problems in their medical record; (2) sensory impairment to pain (such as spinal bifida); (3) identified contact precautions; and (4) previous history of seizures or motion sickness. This study selects 4–12 year-old patients because they experience high level of distress in

venipuncture procedure [4], and distraction is efficacious in this age group [14]. Younger patients will not be included because of their possible limited cognitive and verbal capacity to respond to the questionnaires.

Sample size – Patients

In 2016, about 250 hospitalized patients aged between 4 years to 12 years were admitted to the unit and required venipuncture. Sample size estimation was based on the effect estimated from a previous IVR study using the FPS-R Scale as a primary outcome measure [11]. By using the power analysis software, GPower 3.1, it was estimated that a sample size of 85 participants per group would enable a 2-arm RCT to detect a between-group difference of 0.8 in FPS-R scale with a pooled standard deviation of 1.84 with 80% power at 5% level of significance. Taking into account of up to 15% attrition rate [24], 200 children will be recruited. Furthermore, to account for those who fail in the first attempt of venipuncture, the total sample size is adjusted to 250 children to be recruited with the estimation of 20% of unsuccessful cases in the first attempt. As such, the total recruited number of children will be 250 for the experimental and control groups, with 125 children in each group.

Health care providers

About 10 health-care providers involved in venipuncture procedures (e.g. doctors, phlebotomist, and health care assistants) will be invited to assess their satisfaction for the procedures.

Randomization

Eligible participants will be randomly assigned in a 1:1 ratio to the intervention group who will receive the IVR intervention or a control group that will receive standard care only using stratified permuted block randomization with a block size of 10 to maintain a good balance of participants between the two groups throughout the subject recruitment period. Randomization will be stratified by age group (4–7; 8–12 years old) in equal numbers.

According to Piaget's theory, children from 4 years to 7 years of age belong to the same pre-operational stage, whereas those in the age range 8–12 years belong to the concrete operational stage [31]. Children in different stages differently perceive information and pain stimuli sensitivity [32].

A sequence of grouping identifiers (I=intervention group or C=control group) will be prepared in advance by an independent statistician, using computer-generated random codes for each of the two strata of age group. The group identifiers for each age group will then be put in serially numbered sealed opaque envelopes according to the underlying random sequence list by the statistician. The group allocation of the patients will be assigned according their ages, sequence of enrolment in the study, and the group identifier contained in the corresponding numbered envelopes. Group allocation will be concealed from the research assistant (RA), ward staff, child, and parents/ legal guardians until consent and baseline assessment data has been obtained. Given the nature of the intervention, blinding of participants, RA, and health care provider will be difficult. Nevertheless, the lack of blinding will not necessarily contribute to a source of bias because children are unlikely to change their behavior even when they know they are participating in a certain intervention [33].

Control Group: Standard Care

Participants in the control group will receive standard care without IVR intervention. Standard care includes explaining why and what is being done and saying comforting and supportive words during procedures.

Intervention: IVR

In addition to standard care, children in the intervention group will receive IVR intervention through a commercially available VR headset, which can be fitted into majority of commonly available smartphones. Although other latest headsets, such as Oculus Rift or HTC Vive, can provide high quality of immersion experience, they likewise increase the risk

of contact infection and require a high spectrum personal computer to operate. Therefore, these devices will not be used in this study.

With regard to the IVR modules, previous work conducted by lead PI's research team selected five modules for patients. Examples of these modules include interactive games, cartoon animation, and animated scenery. Among these modules, cartoon animation and interactive games were considered interesting by patients and can capture their attention for an extended period of time. For instances, cartoon animation was preferred by young patients, whereas the interactive game was preferred by adolescents; these results concurred with a previous study [25]. An IVR module that presents procedural information through a customized and child-friendly design more effectively mitigates pain and distress than that with off-the-shelf content [18, 30, 34]. However, these freely downloaded IVR modules that were developed in Western countries with English as the medium may not be completely appropriate for pediatric patients in Hong Kong. In the proposed study, IVR modules have been designed on the basis of suggestions to use age-appropriate modules for patients [16-17] and the experiences of the lead PI, who has successfully developed six cartoon animations in previous studies [35]. The lead PI has produced two developmentally appropriate IVR modules: One for 4–7 year-old children and one for 8–12 year-old children. The two modules share the following common characteristics with those used in previous studies: (1) a wide range of visual and auditory stimuli [11, 23-24]; (2) requiring minimum movement of head and hand to allow the procedures to proceed unhindered [36]; and (3) provision of procedural information. Both modules have been validated by an expert panel and used in pediatric cancer patients during venipuncture.

<u>Module for children aged 4 to 7 years</u>

For children 4–7 years of age, their comprehension of words and sentences is not well-established. However, their curiosity and imaginative thinking are developed [37]. On the

contrary, visual stimulation may be effective in distracting the children [28, 38]. Thus, this VR module presents fast-paced zooming of the screen with cartoon characters enacting various body movements [38]. Pastel tone colors which are less tiring to the eyes are used to provide visual stimulation. The animation "DD is in the hospital" uses simple words and sentences to provide procedural information to the pediatric patients as to why the cartoon character "DD" needs a venipuncture in a child-focused manner. The VR module aims to provide distraction as well as instill a sense of control by exposing patients to the procedure [7, 28, 30].

<u>Module for children aged 8 to 12 years</u>

The main goal of this module is to create an interactive environment for distracting children during venipuncture. The animation will prepare children for venipuncture by providing information, such as (1) why the procedure must be done; (2) what will happen; and (3) how the procedure will feel. The VR module will be followed by an interactive game to increase patients' sense of control over the procedure. In this game, the patients will assist to find out equipment for performing venipuncture in the IVR environment [28, 38].

Implementation Protocol

The venipuncture procedure will be conducted in the treatment room while parents/ legal guardians wait outside. Patients assigned to the intervention group will receive IVR intervention prior to the start of venipuncture until the end of the procedure [11, 23]. The RA will provide simple and standard instruction on how to use the equipment. The headset will then be placed on patient's head and adjusted to ensure a comfortable and secure fit. Patients will be allowed to view the IVR module according to their age group (4–7 years, 8–12 years). During the intervention, the control of the IVR module will only be achieved by the head movement of the patients. The patients will also be told that the intervention will be discontinued if they experience motion sickness, eye discomfort, or headaches.

After receiving IVR intervention, the RA will invite the doctor/phlebotomist to start the venipuncture. The beginning of the procedure is indicated by doctor/ phlebotomist disinfecting the venipuncture site. The RA will note the time of first attempt at venipuncture. The end of the procedure is indicated by application of band aid to the venipuncture site. The RA will remove the IVR equipment from the child after the procedure. The protocol follows for the first venipuncture attempt only. If the first attempt is unsuccessful, additional attempts occurring after the protocol will be completed, but participants will be counted as unsuccessful case of the study.

Fidelity of the Intervention

The fidelity of the intervention will be ensured by recruiting a RA with a minimum of two years of experiences in pediatric care. She will undergo two days' training conducted by the lead PI. The training will include (1) basic knowledge about and application of IVR; (2) procedures of implementing the intervention and collecting data; and (3) management of untoward reactions, such as motion sickness. A minimum of one session conducted by the RA each month will be randomly selected to assess compliance with the implementation protocol by the lead PI. The lead PI and RA will meet monthly to discuss the delivery of IVR intervention and study progress. Feedback will be given accordingly.

Outcome Measures

Primary outcome

Faces pain scale-revised: The FPS-R scale is a 0 to 10 scale comprising six horizontally arranged cartoon faces with expressions of "0 = no pain" to "10 = very painful" [39]. Participants will be asked to point to the face that indicates how much pain she/he feels. The protocol for use of FPS-R is standardized and is a reliable and valid scale for evaluating pain in children [9, 11].

Secondary outcomes

Visual analogue scale for anxiety: A visual analogue scale (VAS) for anxiety will be used to assess the anxiety levels of children aged 4–7 years old. The VAS is a 10 cm horizontal line marked with the words "not worried" (low score) at one end and "very worried" (high score) at the other, with different facial expressions drawn along the line. Children aged between 4 and 7 will be asked to indicate their levels of anxiety by moving a pointer over the line, with higher scores indicating greater anxiety. The VAS is a widely used scale which is reliable and valid for measuring the subjective feelings of children [40]. It has been used previously by PI to assess the anxiety level of children undergoing medical procedures [41].

State anxiety scale for children: The short form of the Chinese version of the State Anxiety Scale for Children (CSAS-C) will be used to measure the anxiety levels of children aged 8-12 [42]. The CSAS-C is a 3-point Likert scale with total scores ranging from 10 to 30. Higher scores indicate greater anxiety levels [41]. The psychometric properties of the short form has been tested and found to correlate strongly with the full form ($r = 0.92$). It has good internal consistency ($r = 0.83$) and convergent validity that differentiate the anxiety state of children under various situations [43]. The PI has previously used it to assess the anxiety level of children undergoing medical procedures with Cronbach's alpha 0.80 to 0.88 [41].

Heart rate: The heart rate of the children will be measured by a standard automatic heart rate monitoring machine (available in the study institution) to assess the physiological responses of children. Heart rate is considered to be objective and definitive in indirectly assessing physiological responses of pain and anxiety of children [23-24].

Salivary cortisol assay: Saliva cortisol assay will be used as to assess the stress levels of the children [44]. The trained RA will collect saliva samples from the patients using the Salivette sampling devices, according to the manufacturer's instructions at 10 min prior and 30 min after the venipuncture procedures [44]. Collected saliva samples will be frozen at −80 °C

until further processing and analysis. Cortisol levels in the saliva samples will be measured using an enzyme-linked immunoassay kit (Salimetrics, PA, USA), according to the manufacturer's instructions.

Length of procedure: A standard stop watch will be used to measure the length of the procedure from the beginning (time when the doctor/phlebotomist starts to disinfect the site) to the end of procedure (time when applies the band aid on the venipuncture site).

Staff satisfaction scale: The staff satisfaction scale will be adopted to measure the satisfaction levels of the health care providers toward the procedure [45]. It consists of 8-item and each rated by a 5-point scale ranging from 1 = strongly disagree to 5 = strongly agree. Higher score presents a higher level of the satisfaction. This scale has been translated by the PI using back-translation method recommended by Brislin (1970) and used in a previous study with the Cronbach's alpha of 0.90 [41].

Cost-effectiveness: Cost analysis will be examined based on incremental cost-effectiveness ratio expressed as incremental cost per every one unit decrease in the primary outcome of the FPS-R scale immediately post-intervention with respect to the baseline. All the cost data involved will be expressed in Hong Kong dollars and valued on the starting date of the study on the basis of non-subsidized cost. All costs incurred will be estimated per each participant using the method of Thompson & Barber [46]. Specifically, the cost of human resources (e.g. doctors, nurses, phlebotomist, and health care assistants) is measured by the salary of the middle rank of the professionals and calculated based on the minutes used to perform the procedures. Costs of consumables to perform the venipuncture (e.g. alcohol pad, glove, butterfly needles, and band aid) will be based on the retail price paid by the finance office of the hospital. In addition, the cost for development of IVR modules and VR goggles will be included in the intervention group.

Data Collection Procedures

Children requiring venipuncture will be identified by the nurse in the pediatric unit. If the children meet the inclusion criteria for recruitment, the nurse will refer the children and their accompanying parents/ legal guardians to the RA in the treatment room, who will give them an information sheet, explain the study and show the IVR equipment. If they agree to participate, written informed consent from the accompany parents/ legal guardians and assent from the children will be obtained. The RA will then acquire socio-demographics information from the parents/ legal guardians and clinical characteristics of the children from medical record before randomization. According to the subject allocation scheme, children in the control group will receive standard care, whereas those in the intervention group will additionally receive IVR intervention.

Participants will be assessed:10 minutes prior to the procedure (T0), during the procedure when the needle insert to the skin (T1), immediately after the procedure indicate by application of band aid to the venipuncture site (T2), and 30 minutes after the procedure (T3). At T0, a set of baseline data - saliva sample for a measure of cortisol, FPS-R, VAS for anxiety, CSAS-C, and heart rate - will be collected from the patients by the RA. At T1, heart rate will be obtained. At T2, FPS-R, VAS for anxiety, CSAS-C and heart rate will be obtained from children. At the same time, the RA will record the length of procedure and invite the health care providers involved to fill in the staff satisfaction scale. At T3, saliva sample for a measure of cortisol, FPS-R, VAS for anxiety, CSAS-C, and heart rate will be obtained from patients again. Please refer to table 1 and figure 1.

Ethical consideration

Ethical approval will be sought from the Research Ethics Committee (Kowloon Central/Kowloon East). Written informed consent from parent/ legal guardians and assent from child will be obtained. Participants and parents/ legal guardians will be informed that the care will not be affected by their participation status.

Data analysis

IBM SPSS 24 will be used for data analysis. Continuous demographic and clinical variables will be presented as their means and standard deviations, whereas categorical data (e.g. sex) will be presented in frequencies and percentages, as appropriate. The intention-to-treat principle will be adopted for the outcome comparisons between the intervention and control groups. The generalized estimating equations (GEE) model will be used to compare each of the outcome measures across the time points between the two groups. GEE model can account for intra-correlated repeated measures data and produce unbiased estimates even if there are missing data, provided that the data are missing at completely random. Cohen's d values will be calculated to estimate the effect sizes of the IVR intervention on the outcome variables. All statistical analyses are two-sided and level of significance will be set at 0.05.

References

1. Walther-Larsen S, Pedersen MT, Friis SM, Aagaard GB, Rømsing J, Jeppesen EM, Friedrichsdorf SJ. Pain prevalence in hospitalized children: a prospective cross-sectional survey in four Danish university hospitals. Acta Anaesthesiologica Scandinavica. 2017;61(3): 328-337.
2. Stevens BJ, Abbott LK, Yamada J, Harrison D, Stinson J, Taddio A, Barwick M, Latimer M, Schott SD, Rashotte J, Campbell F, Finley GA; CIHR Team in Children's Pain. Epidemiology and management of painful procedures in children in Canadian hospitals. Canadian Medical Association Journal. 2011;183(7): E403-E410.
3. Pate JT, Blount RL, Cohen LL, Smith AJ. Childhood medical experience and temperament as predictors of adult, functioning in medical situations. Children's Health Care. 1996;25(4): 281-298.
4. Humphrey GB, Boon CM, van Linden van den Heuvell GF, van de Wiel HB. The

occurrence of high levels of acute behavioural distress in children and adolescents undergoing routine venepunctures. Pediatrics. 1992;90(1 pt 1): 87-91.

5. Kennedy RM, Luhmann J, Zempsky WT. Clinical implications of unmanaged needle-insertion pain and distress in children. Pediatrics. 2008;122(Suppl 3): S130-S133.
6. Noel M, McMurty CM, Chambers CT, McGrath PJ. Children's memory for painful procedures: the relationship of pain intensity, anxiety, and adult behaviors to subsequent recall. Journal of Pediatric Psychology. 2010;35(6): 626-636.
7. Karlsson K, Rydström I, Nyström M, Enskär K, Dalheim Englund AC. Consequences of needle-related medical procedures: A hermeneutic study with young children (3-7 years). Journal of Pediatric Nursing. 2016. 31(2); e109-e118.
8. McMurty CM, Pillai Riddell R, Taddio A, Racine N, Asmundson GJ, Noel M, Chambers CT, Shah V, HELPinKids&Adults Team. Far from "just a poke": common painful needle procedures and the development of needle pain. The Clinical Journal of Pain. 2015;31(10 suppl): S3-S11.
9. Smith RW, Shah V, Goldman RD, Taddio A. Caregivers' responses to pain in their children in the emergency department. Archives of Pediatrics and Adolescent Medicine. 2007;161(6): 578-582.
10. Cohen LL, Blount RL, Panopoulos G. Nurse coaching and cartoon distraction: an effective and practical intervention to reduce child parent and nurse distress during immunizations. Journal of Pediatric Psychology. 1997;22(3): 355-370.
11. Gold JI, Kim SH, Kant AJ, Joseph MH, Rizzo AS. Effectiveness of virtual reality for pediatric pain distraction during IV placement. Cyberpsychology and Behaviour. 2006;9(2): 207-212.
12. Moureau N, Zonderman A. Does it always have to hurt? Premedications for adults and children for use with intravenous therapy. Journal of Intravenous Nursing. 2000;23(4):

213-219.
13. Czarnecki ML, Turner HN, Collins PM, Doellman D, Wrona S, Reynolds J. Procedural pain management: a position statement with clinical practice recommendations. Pain Management Nursing. 2011;12(2): 95-111.
14. Birnie KA, Noel M, Parker JA, Chambers CT, Uman LS, Kisely SR, McGrath PJ. Systematic review and meta-analysis of distraction and hypnosis for needle-related pain and distress in children and adolescents. Journal of Pediatric Psychology. 2014;39(8): 783-808.
15. Bukola IM, Paula D. The effectiveness of distraction as procedural pain management technique in pediatric oncology patients: a meta-analysis and systematic review. Journal of Pain and Symptom Management. 2017;54(4): 589-600.e1.
16. Uman LS, Birnie KA, Noel M, Parker JA, Chambers CT, McGrath PJ, Kisely SR. Psychological interventions for needle-related procedural pain and distress in children and adolescents. The Cochrane Database of Systematic Reviews. 2013;10(10): CD005179.
17. Dahlquist LM, Busby SM, Slifer KJ, Tucker CL, Eischen S, Hilley L, Sulc W. Distraction for children for different ages who undergo repeated needle sticks. Journal of Pediatric Oncology Nursing. 2002;19(1): 22-34.
18. Kuo HC, Pan HH, Creedy DK, Tsao Y. Distraction-based interventions to children undergoing venepuncture procedures. Clinical Nursing Research. [Preprint] 2016.
19. Brown NJ, Kimble RM, Rodger S, Ware RS, Cuttle L. Play and heal: randomized controlled trial of Ditto^{TM} intervention efficacy on improving re-epithelialization in pediatric burns. Burns. 2014;40(2):204-13.
20. Won AS, Bailey J, Bailenson J, Tataru C, Yoon IA, Golianu B. Immersive virtual reality for pediatric pain. Children (Basel). 2017;4(7): pii: E52.
21. Chirico A, Lucidi F, De Laurentiis MD, Milanese C, Napoli A, Giordano A. Virtual reality

in health system: Beyond entertainment. A mini-review on the efficacy of VR during cancer treatment. Journal of Cellular Physiology. 2016;231(2): 275-287.

22. Stanford Children's Health. Hospital-wide access to virtual reality alleviates pain and anxiety for pediatric patients. Available from http://www.stanfordchildrens.org/en/about/news/releases/2017/virtual-reality-alleviates-pain-anxiety?source=whats-new. [Accessed 6th October 2017].
23. Gershon J, Zimand E, Pickering M, Rothbaum BO, Hodges L. A pilot and feasibility study of virtual reality as a distraction for children with cancer. Journal of the American Academy of Child and Adolescent Psychiatry. 2004;43(10): 1243-1249.
24. Wolitzky K, Fivush R, Zimand E, Hodges L, Rothbaum BO. Effectiveness of virtual reality distraction during a painful medical procedure in pediatric oncology patients. Psychology and Health. 2005;20(6): 817-824.
25. Windich-Biermeier A, Sjoberg I, Dale JC, Eshelman D, Guzzetta CE. Effects of distraction on pain, fear, and distress during venous port access and venipuncture in children and adolescents with cancer. Journal of Pediatric Oncology Nursing. 2007;24(1): 8-19.
26. Melzack R, Wall PD. Pain mechanisms: a new theory. Science. 1965;150(3699): 971-979.
27. Lazarus RS, Folkman S. Stress, Appraisal and Coping. New York: Springer; 1984.
28. Jaaniste T, Brett H, Von Baeyer CL. Providing children with information about forthcoming medical procedures: a review and synthesis. Clinical Psychology Science and Practice. 2007;14(2):124-43.
29. De More M, Cohen LL. Distraction for pediatric immunization pain: a critical review. Journal of Clinical Psychology in Medical Settings. 2005;12(4): 281-291.
30. Hoffman HG, Richards TL, Van Oostrom T, Coda BA, Jensen MP, Blough DK, Sharar SR. The analgesic effects of opioids and immersive virtual reality distraction: evidence

from subjective and functional brain imaging assessments. Anesthesia and Analgesia. 2007;105(6): 1776-1783.

31. Piaget J. The origins of intelligence in children. New York: Norton; 1963.
32. Blankenburg M, Boekens H, Hechler T, Maier C, Krumova E, Scherens A, Magerl W, Aksu F, Zernikow B. References values for quantitative sensory testing in children and adolescents: developmental and gender differences of somatosensory perception. Pain. 2010;149(1): 76-88.
33. Silva RD, Austregésilo SC, Ithamar L, Lima LS. Therapeutic play to prepare children for invasive procedures: a systematic review. J Pediatr (Rio J). 2017;93(1):6-16.
34. Miller K, Rodger S, Bucolo S, Greer R, Kimble RM. Multi-modal distraction. Using technology to combat pain in young children with burn injuries. Burns. 2010;36(5): 647-658.
35. Wong CL, Chan CWH, Chan HYL. A Flipped Classroom with Micro-modules in a Foundation Nursing Course. Paper presented at: The CUHK Teaching and Learning Innovation Expo 2016; 2016 December; Hong Kong.
36. Nilsson S, Finnström B, Kokinsky E, Enskär K. The use of virtual reality for needle-related procedural pain and distress in children and adolescents in a paediatric oncology unit. European Journal of Oncology Nursing. 2009;13: 102–109.
37. Wong DL, Hockenberry MJ, Wilson D, Winkelstein ML, Kline NE. Nursing care of infants and children. St. Louis: Mosby; 2003.
38. Wright JA. Animation writing and development: from script development to pitch. (2nd ed.). New York: Focal Press; 2013.
39. Hicks CL, Von Baeyer CL, Spafford PA, van Korlaar I, Goodenough B. The Faces Pain Scale-Revised: toward a common metric in pediatric pain measurement. Pain. 2001;93(2): 173-183.

40. Bringuier S, Dadure C, Raux O, Dubois A, Picot MC, Capdevila X. The perioperative validity of the visual analogue anxiety scale in children: a discriminant and useful instrument in routine clinical practice to optimize post-operative pain management. Anesthesia and Analgesia. 2009;109(3): 737–744.
41. Wong CL, Ip WY, Chan CWH, Kwok MC, Wong PF, Choi KC, Chair SY, Ng KW. The stress-reducing effects of therapeutic play on children undergoing cast-removal procedure. Poster session presented at: The Hospital Authority Convention 2017; 2017 May; Hong Kong.
42. Li HC, Lopez V. Development and validation of a short form of the Chinese version of the State Anxiety Scale for Children. International Journal of Nursing Studies. 2007;44(4): 566–573.
43. Li HC, Wong ML, Lopez V. (2008). Factorial structure of the Chinese version of the State Anxiety Scale for Children (short form). Journal of Clinical Nursing. 2008;17(13): 1762-1770.
44. Patil SJ, Shah PP, Patil JA, Shigli A, Patil AT, Tamagond SB. Assessment of the changes in the stress-related salivary cortisol levels to the various dental procedures in children. Journal of the Indian Society of Pedodontics and Preventive Dentistry. 2015;33(2): 94-99.
45. Tyson ME, Bohl DD, Blickman JG. (2014). A randomized controlled trial: Child life services in paediatric imaging. Pediatric Radiology. 2014;44(11): 1426-1432.
46. Thompson SG, Barber JA. How should cost data in pragmatic randomised trials be analysed? BMJ. 2000; 320: 1197-200.
