# Supplementary material for: Effects of an Immersive Virtual Reality Intervention on Pain and Anxiety Among Pediatric Patients Undergoing Venipuncture: A Randomized Clinical Trial
Source: JAMA Netw Open. 2023 Feb 16;6(2):e230001. doi: 10.1001/jamanetworkopen.2023.0001 (PMC9936341; doi:10.1001/jamanetworkopen.2023.0001)
Supplement: Supplement 2. — eFigure. Screenshots of the Scenarios [file jamanetwopen-e230001-s002.pdf]

Supplemental Online Content

Wong CL, Choi KC. Effects of an immersive virtual reality intervention on pain and anxiety among pediatric patients undergoing venipuncture. *JAMA Netw Open.* 2023;6(2):e230001. doi:10.1001/jamanetworkopen.2023.0001

eFigure. Screenshots of the Scenarios

This supplemental material has been provided by the authors to give readers additional information about their work.

© 2023 Wong CL et al. *JAMA Network Open.*

eFigure. Screenshots of the Scenarios.

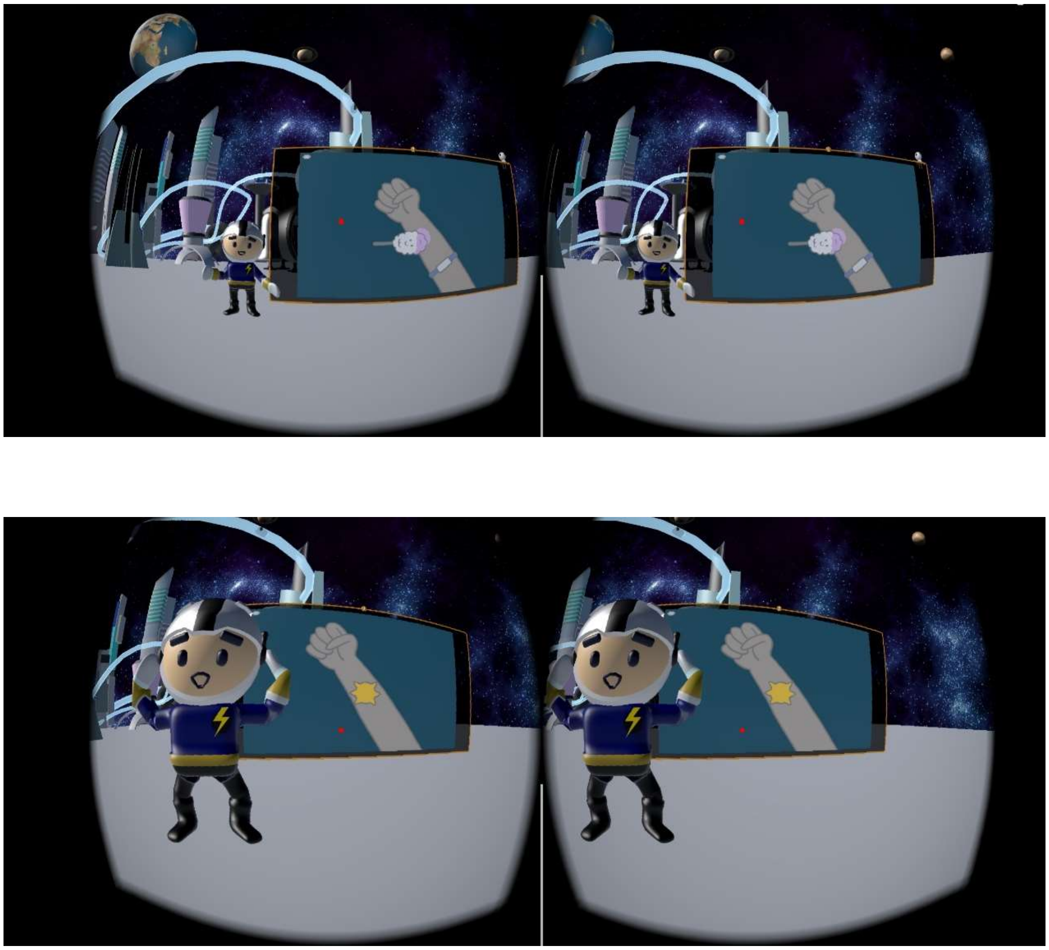

© 2023 Wong CL et al. *JAMA Network Open.*
