# Supplementary material for: Effects of an Immersive Virtual Reality Intervention on Pain and Anxiety Among Pediatric Patients Undergoing Venipuncture: A Randomized Clinical Trial
Source: JAMA Netw Open. 2023 Feb 16;6(2):e230001. doi: 10.1001/jamanetworkopen.2023.0001 (PMC9936341; doi:10.1001/jamanetworkopen.2023.0001)
Supplement: Supplement 3. — Data Sharing Statement [file jamanetwopen-e230001-s003.pdf]

Data Sharing Statement

Wong. Effects of an Immersive Virtual Reality Intervention on Pain and Anxiety Among Pediatric Patients Undergoing Venipuncture. *JAMA Netw Open*. Published February 16, 2023. doi:10.1001/jamanetworkopen.2023.0001

Data
Data available: No

Additional Information
Explanation for why data not available: The data will be shared upon request.
